# Prevalence and Impact of Arrhythmia on Outcomes in Restrictive Cardiomyopathy—A Report from the Beijing Municipal Health Commission Information Center (BMHCIC) Database

**DOI:** 10.3390/jcm12031236

**Published:** 2023-02-03

**Authors:** Haiyan Wang, Sitong Liu, Xilin Zhang, Jianpeng Zheng, Feng Lu, Gregory Y. H. Lip, Ying Bai

**Affiliations:** 1 Department of Neurology and Psychiatry, Beijing Shijitan Hospital, Capital Medical University, Beijing 100038, China; 2 Cardiovascular Center, Beijing Tongren Hospital, Capital Medical University, Beijing 100051, China; 3 Beijing Municipal Health Commission Information Center, Beijing 100034, China; 4 Liverpool Centre for Cardiovascular Science at University of Liverpool, Liverpool John Moores University, Liverpool Heart & Chest Hospital, Liverpool L14 3PE, UK; 5 Department of Clinical Medicine, Aalborg University, DK-9100 Aalborg, Denmark

**Keywords:** atrial fibrillation, bradycardia, ventricular tachycardia, restrictive cardiomyopathy

## Abstract

Background: Data on the outcomes of restrictive cardiomyopathy (RCM) are limited, when the condition is complicated with arrhythmia. This study was designed to investigate the prevalence of atrial fibrillation (AF), ventricular tachycardia (VT) and bradycardia (BC) and their impact on adverse outcomes (intra-cardiac thrombus, stroke and systematic embolism [SSE], heart failure and death) of RCM. Methods and Results: The retrospective cohort study used data collected from the Beijing Municipal Health Commission Information Center (BMHCIC) database from 1 January 2010 to 31 December 2020. There were 745 (64.9%) patients with AF, 117 (10.2%) patients with VT and 311 (27.1%) patients with bradycardia. The presence of AF was associated with an increased risk of SSE (adjusted HR:1.37, 95%CI:1.02–1.83, *p* = 0.04) and heart failure (aHR:1.36, 95%CI:1.17–1.58, *p* < 0.001). VT was associated with an increased risk of intracardiac thrombus (aHR:2.34, 95%CI:1.36–4.01, *p* = 0.002) and death (aHR:2.07, 95%CI:1.19–3.59, *p* = 0.01). Bradycardia did not increase the adverse outcomes in RCM. The results remained consistent and steady when AF, VT and bradycardia were adjusted as competing factors. Conclusions: Cardiac arrhythmia are highly prevalent and associated with adverse outcomes in patients with RCM. AF and VT are more likely to be associated with intracardiac thrombosis, and the presence of AF increased the risk of SSE and HF. The presence of VT increased the risk of death.

## 1. Introduction

Restrictive Cardiomyopathy (RCM) is a rare cardiomyopathy characterized by the abnormal ventricular diastolic function and retained systolic function [1]. Some studies have indicated that RCM may be the least common type of cardiomyopathy [2,3], but the exact prevalence of RCM is unknown. RCM can be divided into primary and secondary RCM. Secondary RCM can be caused by inflammatory invasive diseases such as sarcoidosis and amyloidosis [4,5,6], or occur in late stages of some common heart diseases, such as cardiac hypertrophy, heart cavity dilatation, hypertension, ischemic heart disease and specific cardiac diseases [3,7,8,9,10,11]. In addition, some studies confirmed that RCM can also be acquired through family inheritance. Several RCM-specific mutations have been identified in genes that encode sarcomeric proteins. Among them, cTnI, an important myocardial structural protein, can cause RCM by increasing the calcium sensitivity of cardiomyocytes or by causing changes in genes or proteins that interact with it. Recent studies have also found that mutations in myocardial actin, myosin heavy chain and cTnT genes are associated with RCM [12,13,14,15].

RCM is clinically characterized by arrhythmia, thromboembolism and sudden cardiac death [13]. Arrhythmia, such as atrial fibrillation (AF) and bradycardia (BC) are common in RCM, with one report suggesting a prevalence of AF 74% and ventricular block 19% [16]. Arrhythmia can possibly cause hemodynamic changes, which could be the origin of a thrombus and heart dysfunction. For example, heart failure could develop in the condition of a switch between AF and sinus rhythm due to changes in the conduction system [17]. Patients with RCM often have endocardial thrombosis, which leads to stroke and systematic embolism [18]. Sudden cardiac death can occur in severe cases [19]. While there were some reports on the prevalence of arrhythmia in RCM, their reported impact on the prognosis is generally limited [16].

Therefore, the purpose of this study, using a Beijing population-based registry, was to investigate the impact of arrhythmia on the prognosis, such as stroke and systematic embolism (SSE), heart failure and death in RCM patients.

## 2. Methods

### 2.1. Source of Database

This study used the Beijing Municipal Health Commission Information Center (BMHCIC) database with the whole Beijing population-based registry. The BMHCIC is a mandatory health surveillance and supervision government agency requiring the medical information uploaded from all the 153 hospitals/centers located in the overall Beijing area. The building of the data-set was as previously described [20,21]. The registry covers the demographics information including sex, age, ethnicity, registered date, registered center, contact information, variation of diseases and vital status during each hospitalization and each outpatient visit. The quality of the medical records is guaranteed by periodic supervision and inspection each year (https://www.phic.org.cn/, accessed on 11 May 2022).

The study protocol conformed to the ethical guidelines of the Declaration of Helsinki and was approved by the Ethical committee of the Beijing Tongren Hospital, Capital Medical University. Informed consent was waived due to anonymized and unidentified information for the analysis.

### 2.2. RCM and Atrial Fibrillation, Stroke/Systematic Embolism and Heart Failure

The patients with the first diagnosis of RCM for hospitalization were considered as the baseline and their enrolled date was considered as the start date of the follow-up. Their baseline information was extracted from the case reports accordingly. The patients’ follow-up data since registration were extracted from the revisit of outpatient and re-admission into hospital with endpoints of stroke/systematic embolism, HF and death. The follow-up end time included their last visit date or death date or the end date of the study (31 December 2020). The first-time diagnosis with stroke/systematic embolism, and HF was considered as the date of onset for the condition. Information of death were obtained from the hospitalized case reports or follow-up reports of their relatives.

The patients’ clinical examinations, including 12-lead electrocardiogram (ECG), 24-h Holter monitoring, echocardiography, angiography, and magnetic resonance imaging (MRI) were confirmed by the cardiologists, sonographers and radiologists, respectively. Their diagnosis was confirmed in the BMHCIC registry center using diagnostic codes of *I42.501* for RCM. Only those with RCM confirmed by MRI or cardiac biopsy were included for analysis. Myocardial biopsy was as important as MRI in the diagnosis of restrictive cardiomyopathy because RCM had three stages of pathological changes, which were necrosis, thrombosis and fibrosis.

Therefore, subjects would be included if they met the following inclusion criteria: (1) with available examinations of electrocardiogram and echocardiogram; (2) with RCM confirmed by MRI or cardiac biopsy; or (3) hospitalized for RCM. The exclusion criteria included: (1) suspected of RCM, but not confirmed by MRI or cardiac biopsy; and (2) incomplete medical records.

Arrhythmia included AF, VT and bradycardia. AF was diagnosed if any recordings in the ECG or Holter lasting 30 s or longer. VT was confirmed by ECG or Holter according to the guidelines [22]. Ventricular premature beats and ventricular fibrillation were not included. Bradycardia included sinus node syndrome, atrioventricular block, branch bundle block and ventricular block of any degree.

HF was diagnosed according to guidelines [23] including heart failure with preserved ejection fraction (HFpEF) and heart failure with reduced ejection fraction (HFrEF). Intracardiac thrombus were established according to the results of MRI and echocardiography. Stroke and systematic embolism consisted of stroke which was diagnosed according to the symptoms confirmed by the according imaging.

The composite outcomes included the four separate outcomes (stroke and systematic embolism, HF, intracardiac thrombus and death). 

### 2.3. Statistics Analysis

Continuous variables were expressed as mean ± SD and compared by Student’s t-tests. Categorical variables were expressed as number (percentage %) and compared using Chi-square tests. Unadjusted and adjusted risk of outcomes were obtained by univariable and multivariable Cox regression analysis. Factors in the multivariable analysis included sex, age, hypertension, diabetes mellitus, prior stroke/TIA, enlarged atrium, hepatic cirrhosis, rheumatic disease, all malignancy, old myocardial infarction, anemia, amyloidosis, eosinophilia and sarcoidosis. Kaplan-Meier Analyses were performed to explore the relationship of arrhythmia to the endpoints. SPSS 21.0 (SPSS Inc.; Chicago, IL, USA) was used for the calculation of the study. The *p* value < 0.05 was considered statistically significant. 

## 3. Results

### 3.1. Baseline Characteristics

There were 1148 patients (mean age [SD]: 54.2 [22.1]; 46.1% female) with follow-up information that were diagnosed with RCM by MRI (N = 1042) or myocardial biopsy (N = 106, including 50 patient diagnoses confirmed after heart transplantation) in the Beijing District from 1 January 2010 to 31 December 2020. Among them, 745 (64.90%) patients had AF (age, 60.83 ± 16.47 years), 117 (10.19%) had VT (age, 57.06 ± 16.74 years), and 311 (27.09%) had bradyarrhythmia (age, 57.06 ± 16.74 years). 

As shown in Table 1, RCM patients with AF were more commonly female and had hypertension, diabetes, prior stroke/TIA, OMI, enlarged atrium, malignancy, amyloidosis, anemia, eosinophilia and sarcoidosis. There was an association between RCM patients with VT and anemia (*p* = 0.02), but no significant association with other clinical factors (*p* > 0.10). There was no significant association between RCM patients with bradyarrhythmia and other factors (*p* > 0.10).

### 3.2. The Influence of Type of Atrial Fibrillation on Prognosis

Of the 745 AF patients, 107 patients had paroxysmal AF and 226 patients had non-paroxysmal AF. The rest of AF cases had no recorded AF type. Non-paroxysmal AF had a higher risk of HF and composite outcomes (HF: unadjusted HR: 1.51, 95%CI: 1.18–1.94, *p* < 0.001; composite outcomes: unadjusted HR: 1.33, 95%CI: 1.05–1.69, *p* = 0.02), but similar risk of stroke and systematic embolism, intracardiac thrombus and death (SSE: unadjusted HR: 1.13, 95%CI:0.76–1.68, *p* = 0.55; intracardiac thrombus: unadjusted HR: 1.29, 95%CI: 0.60–2.77, *p* = 0.52; Death: unadjusted HR: 0.47, 95%CI: 0.21–1.06, *p* = 0.07).

### 3.3. Risk of Stroke and Systematic Embolism Related to Arrhythmia

During a median follow-up of 2.17 years (IQR: 0.10–5.58), according to univariable and multivariable Cox regression analysis, RCM patients with AF had a higher risk of stroke and systemic embolism (aHR: 1.37, 95% CI:1.02–1.83, *p* = 0.04). On Cox regression analysis, VT or bradyarrhythmia had no association with the risk of stroke and systemic embolism (VT: aHR:1.41, 95%CI:1.00–1.99, *p* = 0.05; bradyarrhythmia: aHR:1.01, 95%CI:0.78–1.30, *p* = 0.97). 

RCM patients with AF or VT had a greater risk of intracardiac thrombus (AF: aHR: 2.76, 95%CI: 1.57–4.87, *p* < 0.001; VT: aHR:2.34, 95%CI:1.36–4.01, *p* = 0.002). Bradyarrhythmia had no association with intracardiac thrombus on Cox regression analysis (aHR:1.16, 95%CI:0.73–1.85, *p* = 0.52) (Table 2).

**Figure 1:**
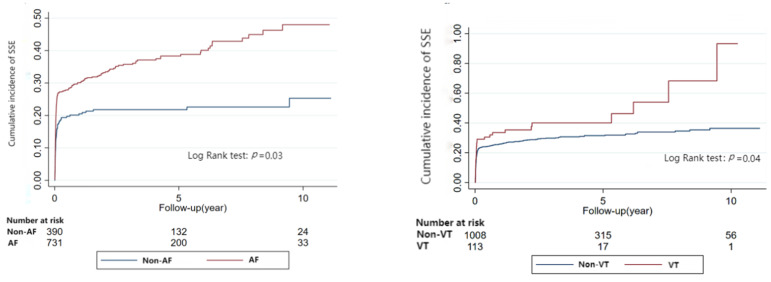
Relationship between arrhythmia and stroke and systematic embolism in restrictive cardiomyopathy (Kaplan-Meier Analyses).

**Figure 2:**
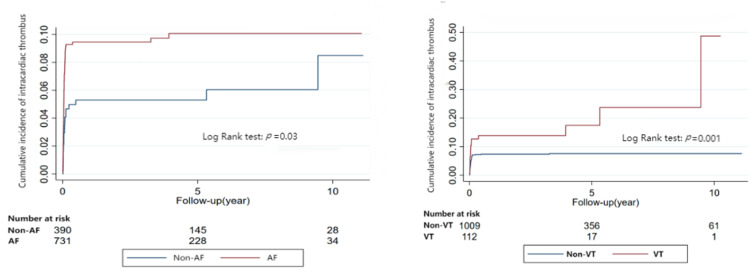
Relationship between arrhythmia and intracardiac thrombus in restrictive cardiomyopathy (Kaplan-Meier Analyses).

All these results were further confirmed by Kaplan-Meier Analyses (Figure 1 and Figure 2 and Supplementary Materials).

### 3.4. Risk of Heart Failure Related to Arrhythmia

**Figure 3:**
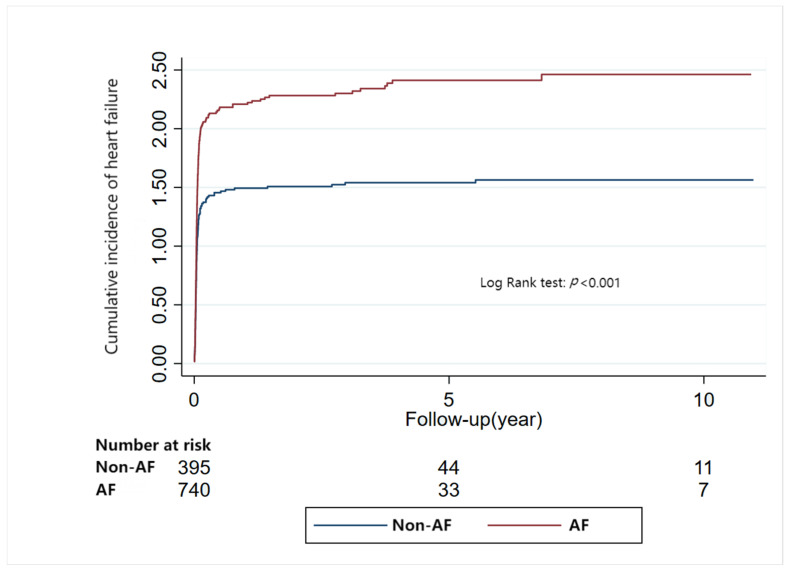
Relationship between atrial fibrillation and heart failure in restrictive cardiomyopathy (Kaplan-Meier Analyses).

During a median follow-up of 0.03 years (IQR: 0.02–0.06), RCM patients with AF had a greater risk of heart failure (AF: aHR: 1.36, 95%CI: 1.17–1.58, *p* < 0.001), while RCM patients with VT or bradyarrhythmia had no association with heart failure (VT: aHR: 1.08, 95%CI: 0.88–1.32, *p* = 0.47; Bradyarrhythmia: aHR: 1.08, 95%CI: 0.94–1.24, *p* = 0.28) (Table 2). The results were further confirmed by Kaplan-Meier Analyses (Figure 3 and Supplementary Materials).

### 3.5. Risk of Mortality Risk Related to Arrhythmia

**Figure 4:**
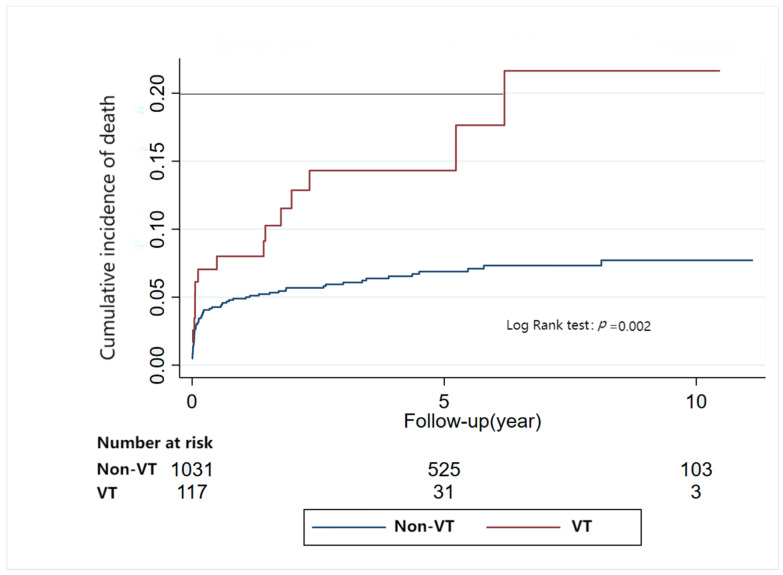
Relationship between ventricular tachycardia and mortality risk in restrictive cardiomyopathy (Kaplan-Meier Analyses).

During a median follow-up of 4.80 years (IQR: 2.44–7.92), RCM patients with VT had a greater risk of death (aHR: 2.07, 95%CI: 1.19–3.59, *p* = 0.01). AF reduced the risk of death in patients with RCM (aHR: 0.51, 95%CI: 0.31–0.83, *p* = 0.01). Bradyarrhythmia had no association with death on logistic regression analysis (aHR: 1.13, 95%CI: 0.71–1.79, *p* = 0.62) (Table 2). The results were further confirmed by Kaplan-Meier Analyses (Figure 4 and Supplementary Materials).

### 3.6. Risk of Composite Outcomes Related to Arrhythmia

**Figure 5:**
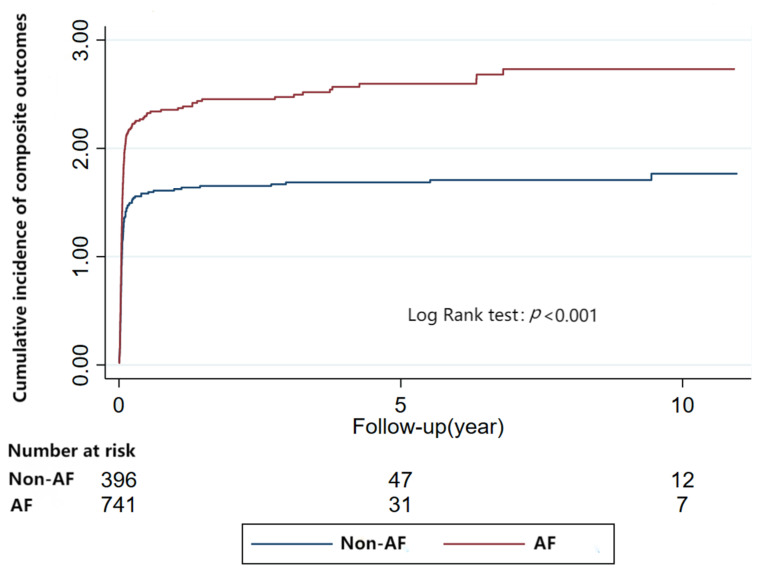
Relationship between atrial fibrillation and composite outcomes in restrictive cardiomyopathy (Kaplan-Meier Analyses).

During a median follow-up of 0.03 years (IQR: 0.02–0.06), RCM patients with AF had a greater risk of composite endpoints (AF: aHR: 1.28, 95%CI: 1.11–1.49, *p* = 0.001), and patients with VT or bradyarrhythmia had no association with composite endpoints (VT: aHR: 1.08, 95%CI: 0.89–1.32, *p* = 0.44; Bradyarrhythmia: aHR:1.06, 95%CI: 0.92–1.21, *p* = 0.43). (Table 2). The results were further confirmed by Kaplan-Meier Analyses (Figure 5 and Supplementary Materials).

By univariate analysis and multivariate analysis, RCM patients with arrhythmias (including AF, VT, and bradyarrhythmia) had a higher risk of stroke and systematic embolism, HF, intracardiac thrombus and composite outcomes (SSE: aHR: 1.55, 95%CI: 1.10–2.17, P= 0.01; HF: aHR: 1.41, 95%CI:1.20–1.66, *p* < 0.001; intracardiac thrombus: aHR: 4.94, 95%CI: 2.34–10.39, *p* < 0.001; composite outcomes: aHR: 1.34, 95%CI: 1.14–1.57, *p* < 0.001). RCM patients with arrhythmia had decreased risk of death after the multivariate analysis (aHR: 0.49, 95%CI: 0.29–0.82, *p* = 0.01) Supplementary Table S2.

## 4. Discussion

Among the RCM patients, cardiac arrhythmia is highly prevalent and associated with adverse outcomes. Most (64.9%) had AF, while 10.2% had VT and 27.1% had bradycardia. AF and VT are more likely to induce intracardiac thrombosis and heart failure; AF raised the risk of SSE, and VT raised the risk of death. 

Similar to previous studies, the prevalence of AF in RCM patients is high [24], possibly due to atrial enlargement [1,25,26,27]. As reported in previous studies [28], we found RCM patients with AF are also more likely to develop heart failure and embolism. Surprisingly, the mortality risk reduced when complicated with AF in RCM patients; similar results were seen in one previous study showing that AF increased the mortality risk in cardiomyopathies, such as dilated cardiomyopathies and hypertrophic cardiomyopathies, but not in RCM. The reason suspected is that RCM patients had more severe cardiomyopathy substrates than patients with hypertrophic or dilated cardiomyopathy, which might cause more severe adverse effects than AF [29].

Different from previous studies, which showed that heart failure and supraventricular tachycardia are common and ventricular arrhythmia are rare [30], the present cohort found a prevalence of 10.2% (N = 117) of VT. The mechanism of VT in patients with RCM is still unclear, and may be related to myocardial amyloid deposition [31] or subendocardial infarction and fibrosis [32]. Of note, VT is associated with increased mortality [33,34,35,36] but heart failure induced by VT doubles the patient’s risk of death with each NYHA grade increase [16]. Surprisingly, VT was not associated with HF in patients with RCM. We speculated that higher success rate of radiofrequency ablation in patients with VT could help reduce risk of HF. In our study, amyloidosis was more prone to occur in patients with VT or bradycardia than AF, possibly due to atrial myocyte less affected by amyloidosis compared to other parts of the heart [37,38,39].

Incidence of intracardiac thrombus was increased in the presence of VT in our study although no significant increase in SSE risks was observed. One speculation is that amyloidosis could damage the endothelium and promote thrombus formation in the heart [30,31]. Of note, ventricular arrhythmia has been a risk marker for embolic embolism in previous studies [40,41,42]. The cause of bradycardia in RCM is not clear, which again may be related to amyloidosis [43] and fibrosis of the conduction system [18,44]. Furthermore, elevated left ventricular diastolic pressure may contribute to the development of an atrioventricular block [18]. In the current study, bradycardia did not contribute to intracardiac thrombosis, heart failure and SSE, but excessive bradycardia may reduce cardiac function and lead to adverse outcomes.

### Limitations

There are several limitations to this study. First, some patients with RCM may not go to the hospital due to lack of obvious symptoms, which may lead to an underestimation of the prevalence. Second, enrolled patients may have other underlying conditions related to the complications of intracardiac thrombosis, stroke and systematic embolism and heart failure, but multivariable factors adjustment performed have reduced the potential effect caused by other reasons. Third, there is little specific treatment for primary RCM, therefore, the data on drug therapies are not always available for these patients.

## 5. Conclusions

Cardiac arrhythmia is highly prevalent and associated with adverse outcomes in patients with RCM. AF and VT are more likely to be associated with intracardiac thrombosis and heart failure, and the presence of AF increased the risk of SSE. The presence of VT increased the risk of death.

## Figures and Tables

**Table 1:** Baseline characteristics of the included patients.

Variables	AF	No-AF	*p* Value	VT	No-VT	*p* Value	Bradycardia	No-Bradycardia	*p* Value
Number	745 (64.90)	403 (35.10)	NA	117 (10.19)	1031 (89.81)	NA	311 (27.09)	837 (72.91)	NA
Female, *n* (%)	361 (48.46)	168 (41.69)	0.03	45 (38.46)	484 (46.94)	0.08	144 (46.30)	385 (46.00)	0.93
Age (y)	60.83 ± 16.47	42.06 ± 25.77	<0.001	57.06 ± 16.74	53.92 ± 22.63	0.07	57.46 ± 20.02	53.04 ± 22.74	0.003
Hypertension, *n* (%)	295 (39.60)	75 (18.61)	<0.001	41 (35.04)	329 (31.91)	0.49	102 (32.80)	268 (32.02)	0.80
Diabetes mellitus, *n* (%)	136 (18.26)	48 (11.91)	0.01	19 (16.24)	165 (16.00)	0.95	47 (15.11)	137 (16.37)	0.61
Prior stroke/TIA, *n* (%)	93 (12.48)	16 (3.97)	<0.001	15 (12.82)	94 (9.12)	0.20	32 (10.29)	77 (9.20)	0.58
Enlarged atrium, *n* (%)	146 (19.60)	58 (14.39)	0.03	19 (16.24)	185 (17.94)	0.65	62 (19.94)	142 (16.97)	0.24
Rheumatic disease, *n* (%)	11 (1.48)	8 (1.99)	0.52	3 (2.56)	16 (1.55)	0.67	5 (1.61)	14 (1.67)	0.94
Hepatic cirrhosis, *n* (%)	20 (2.68)	9 (2.23)	0.64	1 (0.85)	28 (2.72)	0.37	8 (2.57)	21 (2.51)	0.95
All malignancy, *n* (%)	26 (3.49)	26 (6.45)	0.02	5 (4.27)	47 (4.56)	0.89	13 (4.18)	39 (4.66)	0.73
Amyloidosis, *n* (%)	56 (7.52)	80 (19.85)	<0.001	16 (13.68)	120 (11.64)	0.52	40 (12.86)	96 (11.47)	0.52
OMI, *n* (%)	167 (22.42)	63 (15.63)	0.01	21 (17.95)	209 (20.27)	0.55	59 (18.97)	171 (20.43)	0.58
Anemia, *n* (%)	127 (17.05)	35 (8.68)	<0.001	25 (21.37)	137 (13.29)	0.02	51 (16.40)	111 (13.26)	0.18
Eosinophilia, *n* (%)	3 (0.40%)	742 (99.60%)	0.41	1 (0.90%)	116 (99.10%)	0.53	0 (0.00%)	311 (100%)	0.23
Sarcoidosis, *n* (%)	1 (0.10%)	744 (99.90%)	1.00	0 (0.00%)	117 (100%)	1.00	0 (0.00%)	311 (100%)	1.00
NYHA grading, *n* (%)			0.02			0.01			0.17
Class II	23 (4.20%)	21 (9.30%)		0 (0.00%)	44 (6.40%)		8 (3.50%)	36 (6.60%)	
Class III	283 (51.70%)	105 (46.30%)		39 (43.80%)	349 (50.90%)		112 (49.10%)	276 (50.50%)	
Class IV	241 (44.10%)	101 (44.50%)		50 (56.20%)	292 (42.60%)		108 (47.40%)	234 (42.90%)	

NA, not available; AF, atrial fibrillation; TIA, transient ischemic attack; OMI, old myocardial infarction.

**Table 2:** Risk of stroke and systematic embolism in patients with RCM.

Model	AF			VT			Bradycardia		
	HR	95%CI	*p* Value	HR	95%CI	*p* Value	HR	95%CI	*p* Value
Composite Endpoints									
Model 1	1.35	1.18–1.54	<0.0001	1.13	0.93–1.37	0.21	1.08	0.95–1.24	0.25
Model 2	1.28	1.11–1.49	0.001	1.08	0.89–1.32	0.44	1.06	0.92–1.21	0.43
Model 3	1.28	1.10–1.48	0.001	1.06	0.87–1.30	0.54	1.04	0.90–1.19	0.60
Death									
Model 1	0.80	0.51–1.24	0.31	2.34	1.36–4.05	0.002	1.28	0.81–2.03	0.29
Model 2	0.51	0.31–0.83	0.01	2.07	1.19–3.59	0.01	1.13	0.71–1.79	0.62
Model 3	0.49	0.30–0.80	0.30–0.80	2.16	1.23–3.81	0.01	1.02	0.64–1.64	0.94
Stroke and systematic embolism									
Model 1	1.70	1.31–2.21	<0.0001	1.44	1.02–2.02	0.04	1.05	0.81–1.34	0.73
Model 2	1.37	1.02–1.83	0.04	1.41	1.00–1.99	0.05	1.01	0.78–1.30	0.97
Model 3	1.36	1.01–1.83	0.04	1.41	1.00–2.00	0.05	0.98	0.75–1.26	0.85
Heart failure									
Model 1	1.37	1.20–1.57	<0.0001	1.13	0.93–1.38	0.23	1.10	0.96–1.26	0.18
Model 2	1.36	1.17–1.58	<0.0001	1.08	0.88–1.32	0.47	1.08	0.94–1.24	0.28
Model 3	1.35	1.16–1.57	<0.0001	1.05	0.86–1.29	0.62	1.06	0.92–1.22	0.44
Intracardiac thrombus									
Model 1	1.71	1.05–2.79	0.03	2.33	1.37–3.95	0.002	1.13	0.72–1.79	0.59
Model 2	2.76	1.57–4.87	<0.0001	2.34	1.36–4.01	0.002	1.16	0.73–1.85	0.52
Model 3	2.76	1.57–4.86	<0.0001	2.33	1.34–4.05	0.003	1.00	0.62–1.60	1.00

AF, atrial fibrillation; VT, ventricular tachycardia. Model 1. Unadjusted hazard ratio. Model 2. Adjusted factors including sex, age, hypertension, diabetes mellitus, prior stroke/TIA, enlarged atrium, hepatic cirrhosis, rheumatic disease, all malignancy, old myocardial infarction, anemia and amyloidosis. Model 3. Adjusted factors including sex, age, hypertension, diabetes mellitus, prior stroke/TIA, enlarged atrium, hepatic cirrhosis, rheumatic disease, all malignancy, old myocardial infarction, anemia, amyloidosis, VT, AF and bradycardia.

## Data Availability

Date could be shared when requested reasonably.

## Supplementary Materials

The following supporting information can be downloaded at: https://www.mdpi.com/article/10.3390/jcm12031236/s1, Figure S1: Relationship between bradycardia and stroke and systematic embolism in restrictive cardiomyopathy (Kaplan-Meier Analyses); Figure S2: Relationship between bradycardia and intracardiac thrombus in restrictive cardiomyopathy (Kaplan-Meier Analyses); Figure S3: Relationship between arrhythmia and heart failure in restrictive cardiomyopathy (Kaplan-Meier Analyses); Figure S4: Relationship between arrhythmia and mortality risk in restrictive cardiomyopathy (Kaplan-Meier Analyses); Figure S5: Relationship between arrhythmia and composite outcomes in restrictive cardiomyopathy (Kaplan-Meier Analyses); Table S1: Risk of stroke and systematic embolism in different AF types with RCM; Table S2: Risk of stroke and systematic embolism in arrhythmias with RCM.
