# Supplementary material for: Prevalence and Impact of Arrhythmia on Outcomes in Restrictive Cardiomyopathy—A Report from the Beijing Municipal Health Commission Information Center (BMHCIC) Database
Source: J Clin Med. 2023 Feb 3;12(3):1236. doi: 10.3390/jcm12031236 (PMC9917641; doi:10.3390/jcm12031236)
Supplement: Supplementary file 1 [file jcm-12-01236-s001.zip › jcm-2091631-supplementary.pdf]

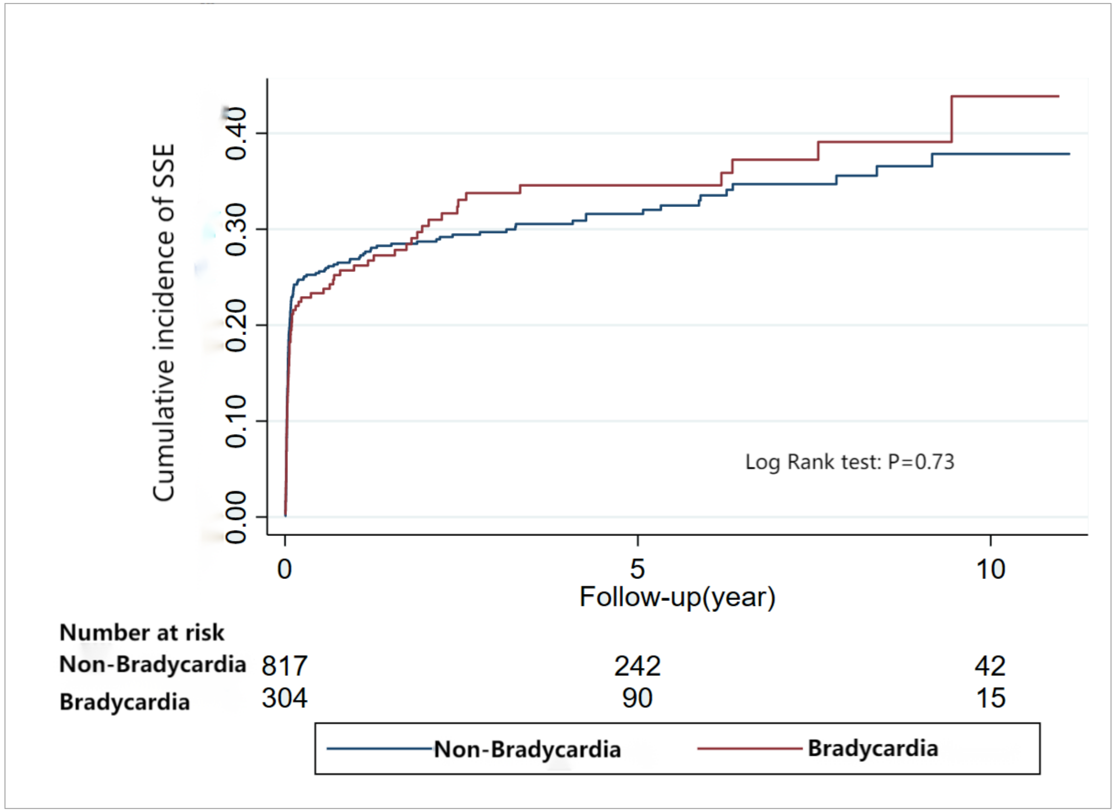

Figure S1. Relationship between bradycardia and stroke and systematic embolism in restrictive cardiomyopathy (Kaplan-Meier Analyses)

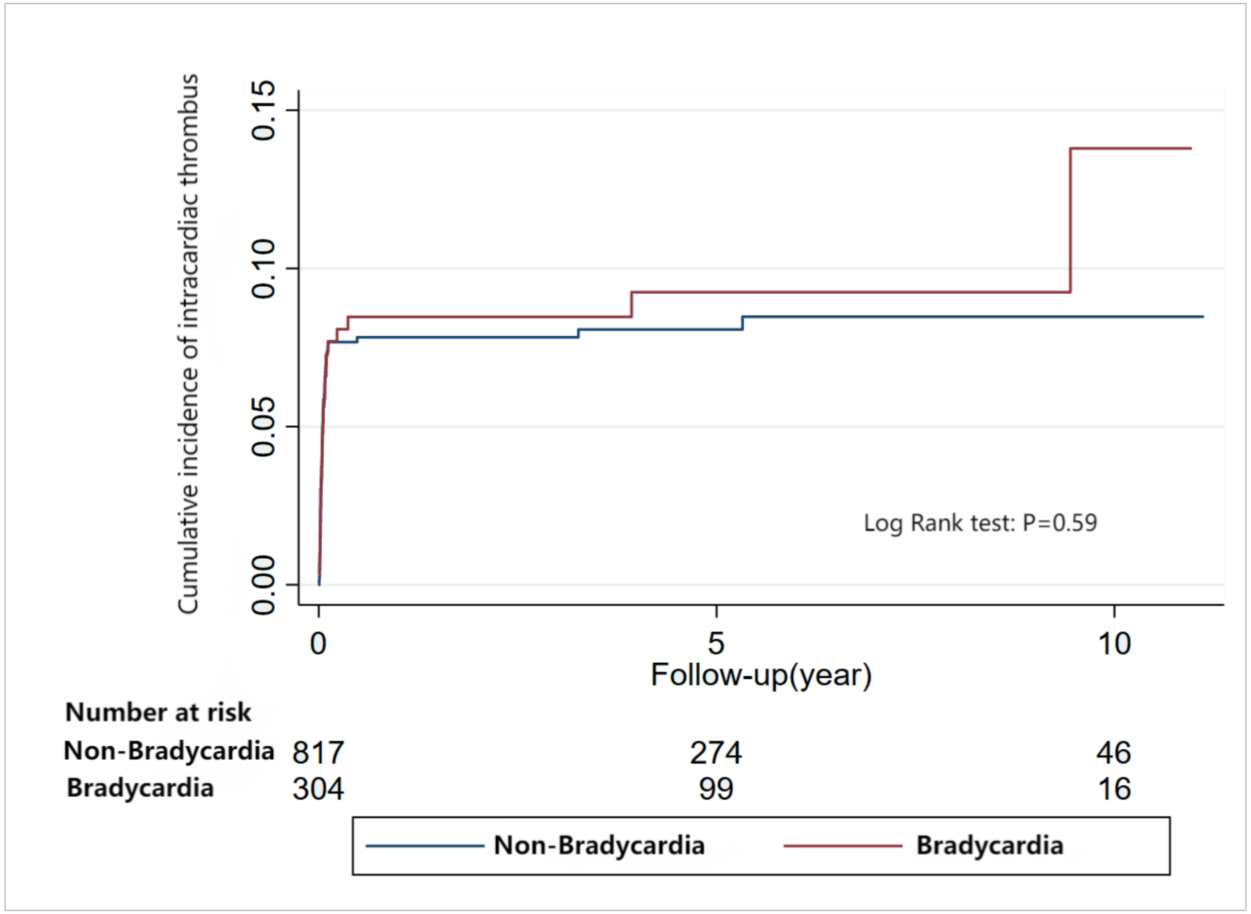

Figure S2. Relationship between bradycardia and intracardiac thrombus in restrictive cardiomyopathy (Kaplan-Meier Analyses)

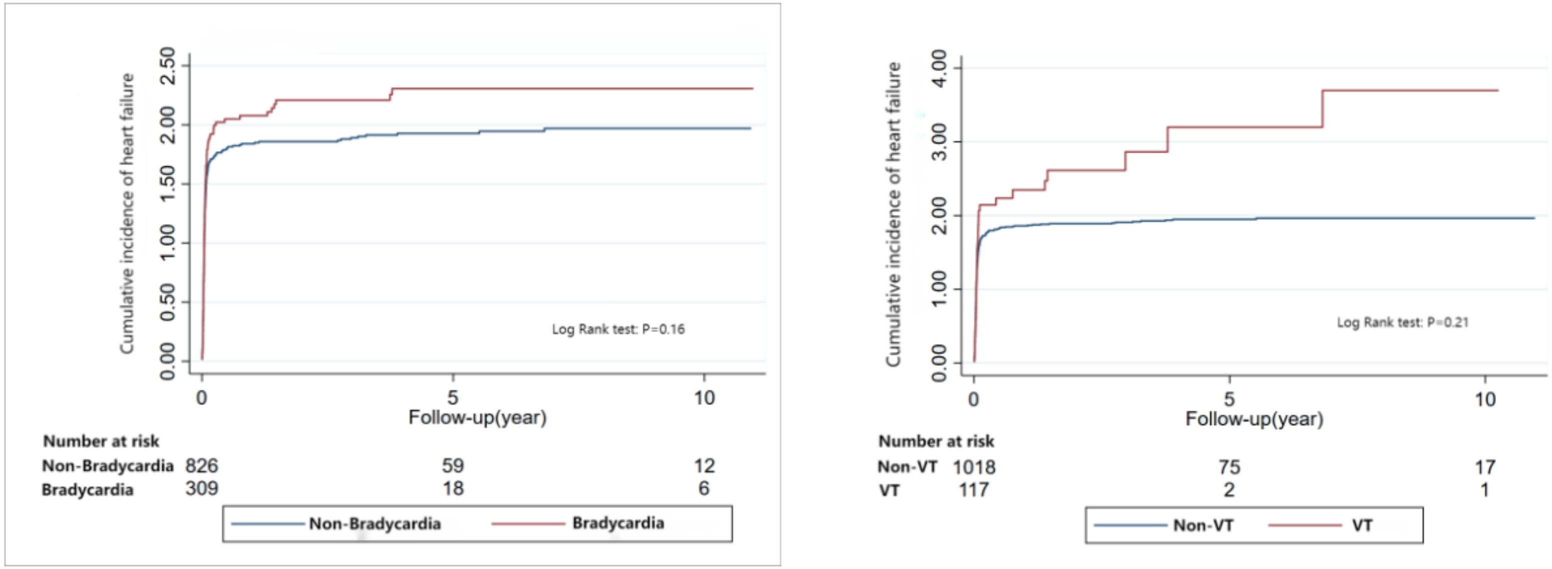

Figure S3. Relationship between arrhythmia and heart failure in restrictive cardiomyopathy (Kaplan-Meier Analyses)

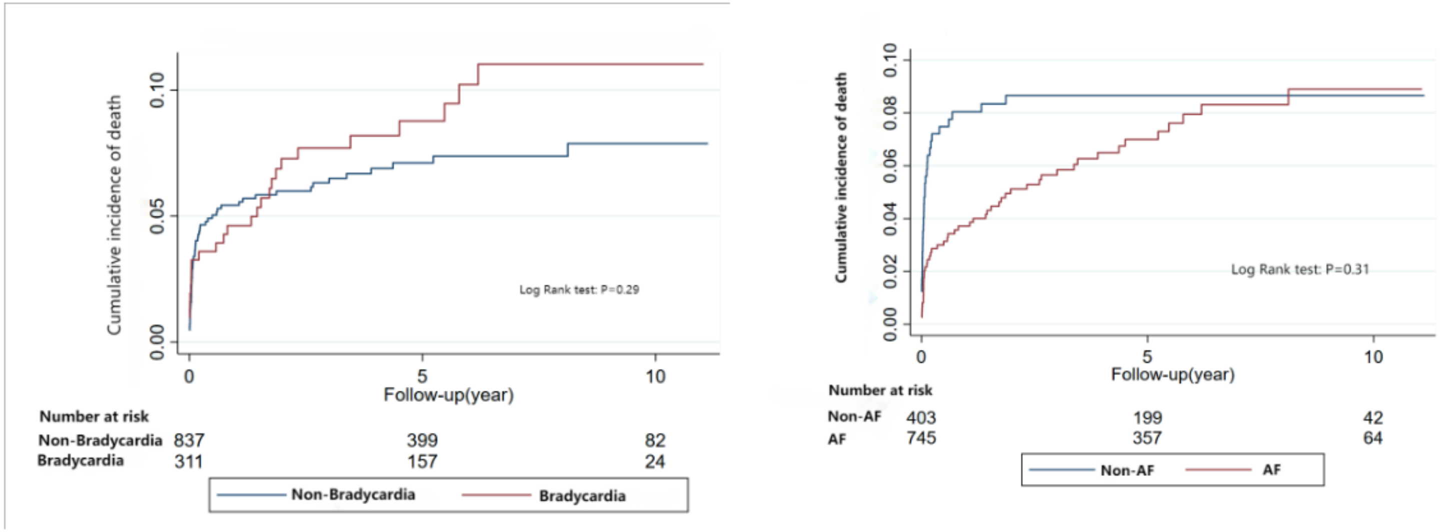

Figure S4. Relationship between arrhythmia and mortality risk in restrictive cardiomyopathy (Kaplan-Meier Analyses)

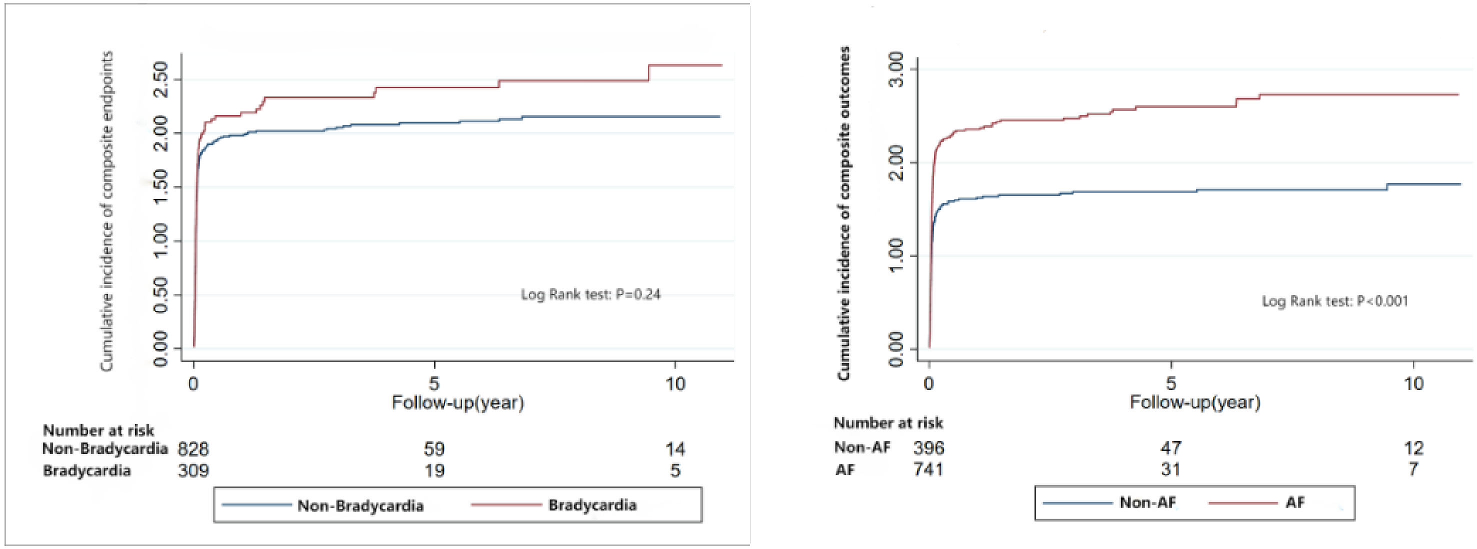

Figure S5. Relationship between arrhythmia and composite outcomes in restrictive cardiomyopathy (Kaplan-Meier Analyses)

Supplementary Table S1. Risk of stroke and systematic embolism in different AF types with RCM.

Model	Paroxysmal and non-paroxysmal atrial fibrillation		
	HR	95%CI	*P value*
Composite Endpoints			
Model 1	1.33	1.05-1.69	0.02
Death			
Model 1	0.47	0.21-1.06	0.07
Stroke and systematic embolism			
Model 1	1.13	0.76-1.68	0.55
Heart failure			

Model 1	1.51	1.18-1.94	<0.0001
Intracardiac thrombus			
Model 1	1.29	0.60-2.77	0.52

Model 1. Unadjusted hazard ratio;

Supplementary Table S2. Risk of stroke and systematic embolism in arrhythmias with RCM.

Model	RCM with arrhythmias		
	HR	95%CI	*P value*
Composite Endpoints			
Model 1	1.45	1.25-1.68	P< 0.001
Model 2	1.34	1.14-1.57	P< 0.001
Death			
Model 1	0.78	0.49-1.24	P= 0.29
Model 2	0.49	0.29-0.82	P= 0.01
Stroke and systematic embolism			

Model 1	1.96	1.43-2.68	P< 0.001
Model 2	1.55	1.10-2.17	P= 0.01
Heart failure			
Model 1	1.46	1.26-1.70	P <0.0001
Model 2	1.41	1.20-1.66	P< 0.001
Intracardiac thrombus			
Model 1	3.01	1.51-6.00	P=0.02
Model 2	4.94	2.94-10.39	P< 0.001

Model 1. Unadjusted hazard ratio;

Model 2. Adjusted factors including sex, age, hypertension, diabetes mellitus, prior stroke/TIA, enlarged atrium, hepatic cirrhosis, rheumatic

disease, all malignancy, old myocardial infarction, anemia, amyloidosis, VT, AF and bradycardia.
